# Anti-social behaviour in the coronavirus pandemic

**DOI:** 10.1186/s40163-022-00168-x

**Published:** 2022-07-04

**Authors:** Eric Halford, Anthony Dixon, Graham Farrell

**Affiliations:** 1 Rabdan Academy, Abu Dhabi, United Arab Emirates; 2 University of Leeds, Leeds, UK

**Keywords:** Anti-social behaviour, Antisocial behavior, Policing, Natural language processing, Artificial intelligence, COVID-19

## Abstract

Anti-social behaviour recorded by police more than doubled early in the coronavirus pandemic in England and Wales. This was a stark contrast to the steep falls in most types of recorded crime. Why was ASB so different? Was it changes in ‘traditional’ ASB such as noisy neighbours, or was it ASB records of breaches of COVID-19 regulations? Further, why did police-recorded ASB find much larger early-pandemic increases than the Telephone Crime Survey for England and Wales? This study uses two approaches to address the issues. The first is a survey of police forces, via Freedom of Information requests, to determine whether COVID-regulation breaches were recorded as ASB. The second is natural language processing (NLP) used to interrogate the text details of police ASB records. We find police recording practice varied greatly between areas. We conclude that the early-pandemic increases in recorded ASB were primarily due to breaches of COVID regulations but around half of these also involved traditional forms of ASB. We also suggest that the study offers proof of concept that NLP may have significant general potential to exploit untapped police text records in ways that inform policing and crime policy.

## Introduction

The COVID-19 pandemic brought dramatic changes to recorded crime patterns and trends in many countries (Abrams, 2021; Andresen & Hodgkinson, 2020; Ashby, 2020a, 2020b; Borrion et al., 2020; Dai et al., 2021; Hoehn-Velasco et al., 2020; Estévez-Soto, 2020; Gerell et al., 2020; Campedelli et al., 2020; Andresen & Hodgkinson, 2020; Nivette et al., 2021; Payne et al., 2020, 2021; Piquero et al., 2020; Wang et al., 2021). For the most part, lockdowns and social distancing restricted the movement of people in ways that disrupted and caused sharp declines in recorded crime types. In England and Wales there were rapid declines in theft, robbery, violence and sex offences, shoplifting, bike theft, vehicle crime, and burglary, particularly in inner city and town centre areas (Office for National Statistics, 2020, 2021a, Halford et al., 2020, Langton et al. 2021a, b).

**Fig. 1:**
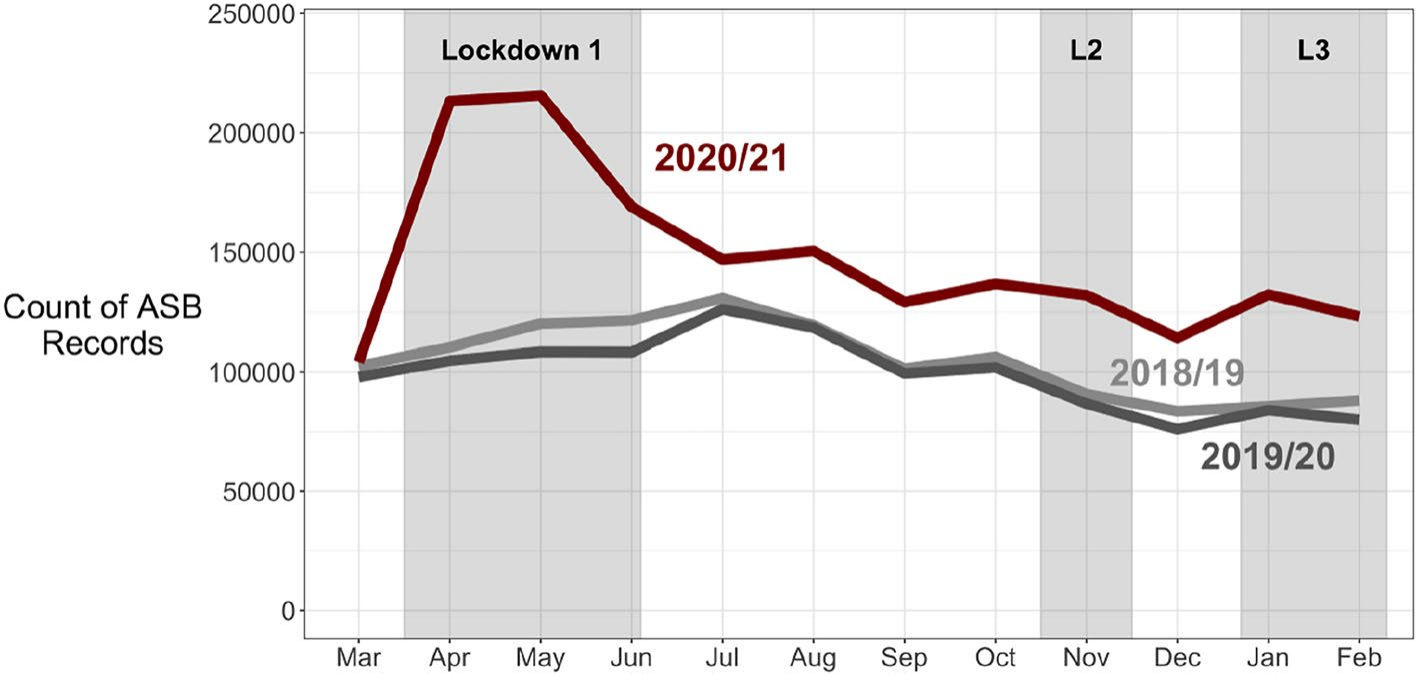
Monthly police-recorded ASB in England and Wales

**Fig. 2:**
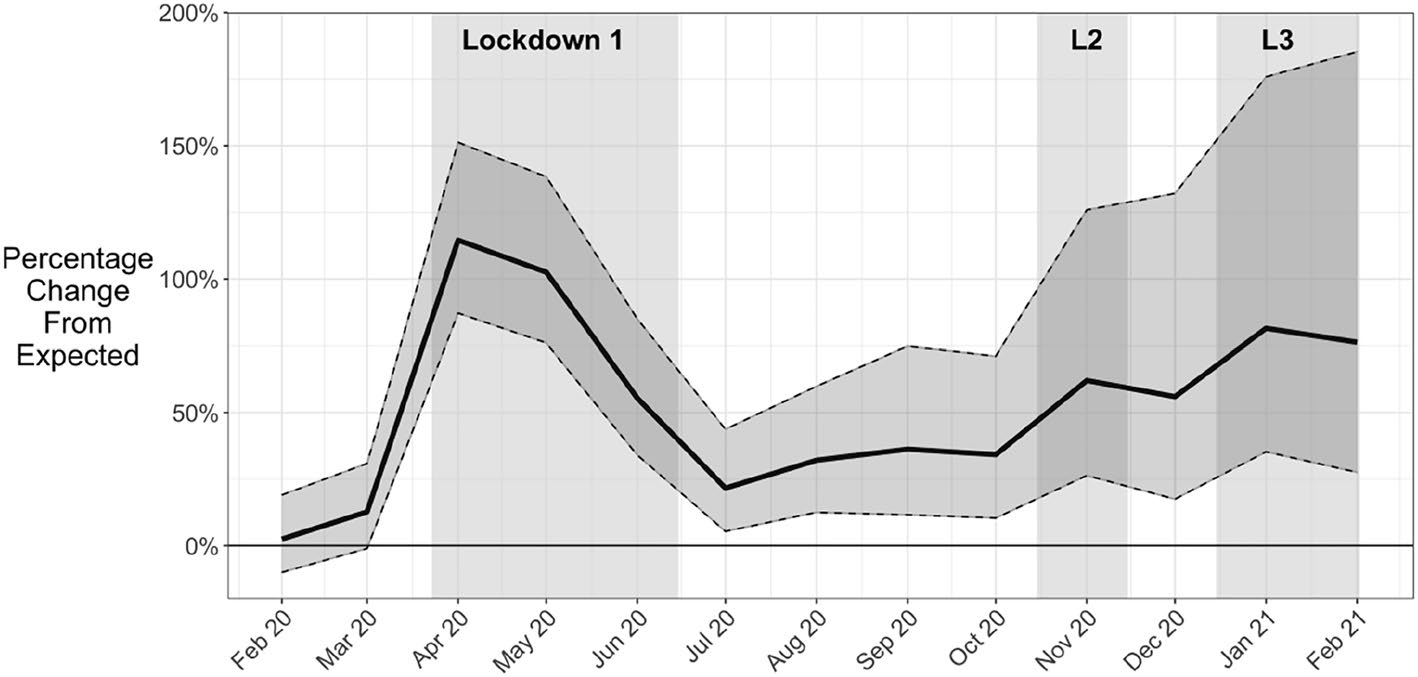
Percent difference between observed and expected recorded ASB (shaded = 95% confidence intervals

In stark contrast to the general declines in recorded crime, the rate of recorded anti-social behaviour (ASB) in England and Wales rose sharply to more than double its expected level in the early months of the pandemic. It remained statistically significantly above expected levels across 2020 and the first quarter of 2021 (Figs. 1 and 2). However, the Telephone Crime Survey for England and Wales (TCSEW) indicated more modest early-pandemic ASB increases (described further below). These empirical features identify our research questions. Why was recorded anti-social behaviour so different? Why did it spike upwards when most recorded crimes declined?

In what follows, for simplicity we tend to refer to ‘traditional ASB’ for all ASB except for breaches of COVID-19 regulations, and to ‘COVID-regulation-breach ASB’ or similar for breaches of COVID-19 regulations (detailed further below) that were recorded by police as ASB. 

This study uses two main approaches to shed light on the anomalous ASB trend of the pandemic. Both approaches are novel. The first is an original survey of how ASB was defined by police, undertaken via Freedom of Information (FOI) requests submitted to all regional police forces in England and Wales. The second is the use of natural language processing (NLP), a form of artificial intelligence combining computational linguistics and statistical learning models, to interrogate the text of a sample of police ASB records. To our knowledge this is the first usage of NLP to interrogate police records in this fashion, and then we discuss the broader implications later.

The survey of police forces finds differences between police forces in whether or not they recorded COVID-regulation-breaches as ASB. It finds that police force areas with the largest increases in ASB were mostly, but not always, those that included COVID-regulation breaches of ASB. Hence the survey offers evidence that the increase in recorded ASB was likely to be primarily, but not solely, due to the inclusion of breaches of COVID regulations.

The NLP analysis of the text of a sample of police incident records indicates that half of the above-expected increase in ASB were solely breaches of COVID regulations, and the remainder were incidents where both a breach of COVID regulations occurred alongside a traditional form of ASB (such as a noisy party which was also a gathering of size exceeding regulations).

Note that both approaches produced similar findings, and that that both found some increase in the rate of recorded ‘traditional’ ASB, which itself is contrary to what occurred for most recorded crime types. However, since the TCSEW gauged breaches of COVID regulations separately from ASB, this explains the large part of the differences between the police records and the survey. Later we discuss why there may have been an increase in traditional ASB.

Much of this study is concerned with methodological details, in which we include a discussion of limitations. Following a further introduction to ASB to set the scene, the description of data, method and results is followed by discussion and conclusions.

### What is ASB?

A recent parliamentary Briefing Paper notes two types of ASB, such that:

- “ASB that occurs within a housing context is defined as behaviour that causes or is likely to cause ‘nuisance or annoyance’
- ASB that occurs in public spaces is defined as behaviour that causes or is likely to cause ‘harassment, alarm or distress.” (Brown & Sturge, 2020; 6).

ASB can impose significant harms on victims and costs to society (Heap 2016, 2020). A range of civil remedy sanctions is applied to address ASB including civil injunctions, Community Protection Notices, and Criminal Behaviour Orders as outlined by the Anti-social Behaviour, Crime and Policing Act 2014.^1^ There is a fascinating history of ASB and the legislative, policing and others responses including the period of Anti-social Behaviour Orders (ASBOs) that preceded current tools, though this is not our main focus here (Adams & Millie, 2021; Burney, 2002, 2009; Cornford, 2011; Harradine et al., 2004; Millie et al., 2005a, 2005b).

Typical examples of traditional ASB are ‘noisy neighbours, vandalism, graffiti, public drunkenness, littering, fly-tipping [illegal waste disposal], and street drug dealing’ (Brown & Sturge, 2020; 6; see Harradine et al., 2004 for a list). In the definition of ASB, the broad parameters set by terms such as ‘likely to cause’, ‘nuisance or annoyance’ and ‘distress’ means that interpretation of potential ASB, both by the public and police, can be subjective and contentious. Simply put, most legally proscribed behaviour is more closely defined. However, the flexibility of ASB’s definition is intentional because it serves as a broad brush to sweep up many unwanted behaviours that are difficult to otherwise categorise and where illegality is uncertain. The definitional flexibility can be an advantage when the set of potential remedies provides leverage to police and other agencies without resorting to criminal sanctions. This saves time and resources and avoids assigning criminal records to offenders. Problems have arisen, however, because ASB sanctions can quickly lead to criminalisation if an order is breached. For example, if a noisy neighbour breaches a Criminal Behaviour Order, then that breach is a criminal offence. Along with the fact that the effectiveness of the sanctions in curtailing ASB is questionable, this defines one of the most contentious areas of ASB policy (Adams & Millie, 2021; Burney, 2009; Cornford, 2011; Harradine et al., 2004; Millie et al., 2005a, 2005b). The definitional ambiguity of ASB can also be a disadvantage when there is disagreement between parties over whether or not behaviour is problematic. Whether a party is noisy and annoying depends not just on the noise level but on contingent factors including frequency of occurrence, time of day, day of week, proximity to others, type and tempo of noise, and the circumstances of those involved. Adults who need to be up early for work, for example, or parents with sleeping children, may be less forgiving. Likewise, a one-off noisy party for a special occasion where neighbours have been forewarned, permissions asked, or invitations issued, might be ignored and forgiven. However, a party of the same volume could be interpreted as ASB if it is unexpected, and particularly when repeated: repetition is a particularly damaging ASB feature (Heap, 2021). This hypothetical example is limited to noisy neighbours, and when the diverse nature of potential ASB and contexts is considered, the permutations are effectively infinite.

When police officers decide whether or not a complaint is recorded as ASB, however, their decisions are constrained by national and regional force-level guidance (Wooff, 2015). The guidance issued by individual forces was, as we show below, particularly important in the coronavirus pandemic.

The relationship between ASB and other offence types should also be considered. ASB is sometimes used to record less serious forms of incidents that are otherwise recorded as public order offences. Conceivably, then, changes in policing priorities could cause a shift in recording from one to the other if there was a change in the threshold for recording a public order crime. While this cannot be entirely ruled out here, public order crime trends in the pandemic were more similar to those of other recorded crime types than to ASB (Dixon et al. 2022), and we have no particular reason to expect a threshold-change accounts for the main ASB trends, while we are able to offer an evidence-based alternate explanation.

### Pre-pandemic ASB

ASB in England and Wales experienced a peak in the early 2000s but had been in decline for close to two decades before the pandemic. Between 2009 and 2019, ASB incidents recorded by police had declined over 60 percent, from 3.7 million incidents in 2009 to 1.4 million in 2019 (ONS, 2019). The Crime Survey for England and Wales (CSEW) gauges seven categories of ASB witnessed by respondents in their local area, from which a composite measure of ‘high-level ASB’ is generated.^2^ All CSEW measures concur that ASB was in long-term decline, but with some variation by type of behaviour.^3^ The two most prevalent local ASB problems reported in 2020 were ‘rubbish or litter lying around’, and drug dealing, witnessed by around a quarter of respondents, but down from around a third in the early 2000s. In 2020, between 12 and 14 percent of respondents reported witnessing drunk or rowdy people in public places, teens hanging around on the street, or property damage (including vandalism and graffiti): all three of these ASB categories had declined by about two-thirds from the early 2000s when they were witnessed by around a third of respondents.

The ASB category found by the CSEW to exhibit the most dramatic decline this century was abandoned or burnt-out cars, witnessed by a quarter of respondents in the early 2000s but by two percent in 2020. Aside from this, of the seven ASB categories gauged by the CSEW, ‘noisy neighbours or loud parties’ was least prevalent. Experiencing noisy neighbours as ASB was typically around 10 percent, peaking at 12 percent of respondents in the early 2010s, declining to 8 percent by 2020: a 50 percent decline in the pre-pandemic decade. Hence generally speaking, in the year before the pandemic, the level of ASB experienced locally in England and Wales was at its lowest levels for at least 20, and probably for more than 30 years. In what follows, when estimating the ASB rate that would be expected if the pandemic had not occurred, we account for this long-term trend in our ARIMA statistical models that are described further below.

### Lockdown laws: COVID regulations

In mid-2020, the UK population was 67 million^4^ residing in England (84.3 percent), Scotland (8.1 percent), Wales (4.7 percent), and Northern Ireland (0.64 percent). Separate but broadly similar regulations were introduced across UK countries, summarised in a House of Commons Briefing (Barber et al., 2021)^5^ and given broad-brush treatment here for brevity’s sake.

The first national stay-at-home lockdown was introduced on 26 March 2020 following a week of recommendation cessation of non-essential travel. Schools and non-essential businesses were closed and, aside from key workers, people were required to stay home for all but essential shopping. Face-masks and two-meter social distancing were required indoors when outside the home. One hour of local outdoor exercise per day was allowed and, from May 2020, it was legal to meet outdoors with one other person. The end of first lockdown is typically dated to the end of June, with pubs re-opening in the first week of July.

Summer 2020 brought lower infection rates, reduced travel restrictions and the policy ‘Eat Out to Help Out’ which subsidised restaurant prices to assist the ailing hospitality industry. Face-mask requirements and two-meter social distancing continued. With the resurgence of COVID-19 rates in late summer the ‘rule of six’ restricted gatherings to six persons from 14 September,^6^ and England’s second national stay-at-home lockdown was introduced on 04 November 2020. It was less restrictive than the first, with schools remaining open, ‘support bubbles’ allowing single adult households to mix with one other household, and unlimited outdoor exercise (Lawrie, 2020). Second lockdown was rescinded on 02 December 2020 for the December holiday period but the third national lockdown, with schools remaining closed after the holiday, began on 06 January 2021. Schools re-opened on 08 March 2021 and non-essential businesses on 12 April 2021, although indoor mixing of different households remained prohibited. All COVID-related restrictions in England were removed on 24 February 2022 with the publication of the ‘Living with COVID’ plan.^7^

### What would we expect to happen to ASB during the pandemic?

The primary theoretical reference point here is the lifestyle theory of victimization (Hindelang et al., 1978, Maxfield, 1987) translated into what Halford et al. (2020) offer as a mobility theory of crime in the pandemic (to which we would add ASB). This suggests that ASB will change in proportion to changes in the movement of people in different locations. ASB is concentrated in residential areas (recall that the CSEW measures of ASB relate to experience in and around where respondents live), and so, other things equal, this would be the primary area of interest. This locational difference distinguishes ASB from many types of recorded crime that occur in and around public transport, workplaces, retail and entertainment areas where the movement of people declined dramatically and caused crime to decline.

Different effects would be expected for different types of ASB, and to vary by lockdown according to the specifics of the regulations. Generally speaking, in stay-at-home lockdown, we might expect an *increase* in ASB between neighbours because of the increased residential population and an increase in opportunities for friction between neighbours to occur. However, to the extent that regulations were adhered to, we might expect a decrease in the occurrence and witnessing of rubbish/litter lying around, drug dealing, people bring rowdy in public places, teens hanging around, property damage, and abandoned or burnt-out cars, because people were not allowed out and about on the street. For present purposes, however, we do not have an empirical handle on these different ASB types in the police records.

### ASB in the pandemic

There is a dearth of research into ASB during the pandemic. A survey-based study of neighbour disputes during the pandemic in 70 Mexican cities identified a 42 percent increase in noise nuisance, an increase in ‘unruly children’ of 35 percent, increased littering and garbage disputes of 32 percent, and increased ‘gossip or misunderstandings’ of 19 percent. (Hoehn-Velasco et al., 2020). A self-report study in the United States found, after controlling for a range of sociodemographic variables, that ‘individuals reporting high levels of antisociality engage in fewer social distancing measures’ (O’Connell et al., 2020; p. 2). A study on online antisocial activity indicated increased sinophobic sentiment (Schild et al., 2020) and increased hate speech directed at vulnerable groups as well as xenophobia (Awal et al., 2020). While these studies do not necessarily use the same definition of anti-social behaviour referred to here, they give a useful indication of related research.

A briefing that analysed police-recorded ASB in England and Wales demonstrated the statistically significant increase relative to expected levels in first lockdown (Dixon et al. 2020). It found the largest relative increases in police forces around south Wales and London, that the percentage increase in area-based ASB was inversely related to previous ASB levels (that is, ASB increased most in percentage terms in those areas which previously had least ASB), and that recorded ASB increases in police force areas were for the most part not strongly correlated with changes in public order offences or the rate of fixed penalty notices.^8^

The telephone CSEW (CSEW, Office for National Statistics, 2020) asked respondents about experiences in first lockdown. The telephone CSEW was the practical version of the CSEW undertaken during the pandemic when face-to-face interviews were not possible. Crime victim surveys are methodologically preferable to police records for many purposes because of their representativeness and consistency. One in five adults reported to the telephone CSEW that ASB was a problem in their local area, two third that ASB levels were about the same as before, and a fifth thought ASB had declined. However, 14–18 percent thought ASB had increased in first lockdown, which appears to contradict the much larger increase identified in police records. Note also, however, that 51 percent of adults responding to the TCSEW reported witnessing breaches of COVID regulations during first lockdown.

## Data and method

### Police records

The police-recorded ASB data was publicly available, open source.^9^ Monthly counts by police force were generated and missing months imputed as an average of neighbouring months. The level of ASB recorded during the pandemic is here compared to expected levels.

Expected national rates of recorded ASB were estimated using the ARIMA time-series modelling approach regularly used to estimate excess death rates in the pandemic, and previously applied to pandemic crime (Ashby, 2020a, 2020b; Estévez-Soto, 2020; Halford et al., 2020). Time series modelling can be more accurate than year-on-year comparisons because it accounts for longer-term underlying trends and month-to-month relationships that are not replicated from year to year. We refer to national trends but were obliged to omit Greater Manchester Police and West Mercia police forces due to lack of data availability. Five years of monthly recorded ASB counts from March 2015 to January 2020 were used as the baseline, and the modelling undertaken in R with the Hyndman (2021) forecast package. The model produced point forecasts of expected ASB with 95 percent confidence intervals that reflect uncertainty.

At the police force level, monthly percentage changes were calculated as the difference from the same period in the previous year. This was because, with the relatively smaller areas and numbers involved, the normality assumptions required for ARIMA did not always hold. Lockdown periods in 2020/21 were compared against the same period in 2019/20 at the police force to calculate percentage change.

### Survey of police recording practice

FOI requests are widely used in research (Luscombe and Walby, 2020; Savage & Hyde, 2014; Worthy et al., 2017). Government and some public organisations have a statutory duty to release information they hold, on request by a member of the public, unless precluded by caveat. FOI requests are written submissions, typically by email, that can only request information already held. Our FOI request was sent to all 43 territorial police forces in England and Wales. It sought to identify whether individual forces included breaches of COVID-regulations in their ASB records. To reduce the possibility that the request was misunderstood or misinterpreted, it contained an explanatory paragraph plus nine questions (Table 1) probing the issue from different angles (see Appendix 1 for the full request).

**Table 1:** Questions included in FOI request

Number	Question
1	Generally, during the pandemic how have you recorded within the control room incoming reports relating to COVID-19 breaches?
2	How did this recording practice manifest, either directly or indirectly, to data.police.uk reporting?
3	Did your force record reports of COVID-19 rule infringements as ASB?
4	Did your Force use an existing category for COVID-19 rule infringement other than ASB?
5	Did your force record reports of COVID-19 rule infringements as another category that is reported to data.police.uk?
6	If you did use an alternate incident class such as ASB, did you apply any ‘tagging’ system to capture the COVID infringements within recorded incidents?
7	If you did use a tag what tag labels did you use?
8	Did your force create a new category for recording reports of COVID-19 infringements?
9	If so is this new category reportable to data.police.uk?

We received responses from 37 forces, a response rate of 86 percent. Responses were graded independently by two research team members. Response clarity was assessed (as ‘unclear’, ‘mostly Clear’ or, ‘certain’). The substantive response was categorised to determine whether COVID-regulation breaches were recorded as ASB and whether there was a subsequent change in recording protocol. Where a clear picture of recording practice could not be ascertained, it was recorded as not known.

### Natural language processing of ASB incidents

While it has previously been suggested that NLP might be used to inform policing (Dixon & Birks, 2021), there is not, to our knowledge, a previous application such as the one undertaken here. NLP was undertaken on the text information contained in police incident logs. In essence, the NLP was an automated means of checking the text content of incident logs to check whether or not each log was traditional ASB, a breach of a COVID-regulation, or a combination of the two.

The NLP was a time-consuming undertaking and so, for practical purposes, used all incidents recorded as ASB across the 13 months from 1 January 2020 to 31 January 2021 for a single force, comprising 93,809 incident logs. Force practice was to include breaches of COVID regulations as ASB, the force ASB trend generally tracked the national ASB trend (that is, with a major increase in recorded ASB during the first national lockdown), and the force-level rate of divergence from expected ASB levels recorded for each lockdown was close to the median for forces that included COVID-regulation beaches as ASB. Additional research with other forces would be required to more firmly establish national representativeness, but it is reasonable to expect that the analysis sheds useful light on what also happened in other forces that included COVID-regulation breaches as ASB.

Each incident log included a ‘tag’, applied in-house by call handlers, which distinguished ‘Specific COVID Complaint’ incidents from ‘Traditional ASB’. The tag had been requested by Operation Talla, the national police operation during the pandemic. For an incident log entry to be classed as a Specific COVID Complaint there needed to be a specific reference to the nature of the breach of lockdown law. For example, during the time when gatherings of six people were permitted, an incident noting that ‘There were 5 people in the garden’ would not be a Specific COVID Complaint while ‘There were 5 people in the garden, and none of them were social distancing’ would be a Specific COVID Complaint. The Traditional ASB category focused on whether, in the absence of any COVID complaint there was still an ASB element. For example “There is a garden party with at least three families, and they are not from the same bubble” would not be considered Traditional ASB, but “There is a garden party with at least three families, they are not from the same bubble, and the noise has been going on for some time” would be categorised as both a specific COVID Complaint and Traditional ASB.

NLP models were built using the Longformer transformer architecture (Beltagy et al., 2020), a pre-trained language model designed for extended pieces of text. The NLP models were trained for the present study using data labelled by two independent researchers, with ties adjudicated by AD. Validation data was used to tune the hyper parameters that produced the final model. Assessment of the final model was conducted on test data (200 randomly selected incidents) not previously seen by the model, and the metrics are shown in Table 2.

**Table 2:** Metrics for final NLP models

Metric	‘Specific COVID Complaint’ model	‘Traditional ASB’ model
Accuracy	90%	92%
F1	0.80	0.96
Matthews correlation coefficient	0.74	0.68

F1 and Matthews correlation coefficients used because they are better measure when the datasets are imbalanced between classes

Once built, the model was used to classify all ASB incidents for the 13 months. The labelled data was aggregated to monthly counts and these subdivided to determine whether they were likely Traditional ASB, likely COVID Specific Complaints or whether they were a mix of the two. Our machine-derived COVID label was then combined with the COVID tag to produce a final classification of COVID-related ASB incidents. Further details on the NLP methodology is included as Appendix 2.

For the discussion of findings below, the NLP-derived estimates are compared to expected ASB levels for the force. Expected ASB was estimated using ARIMA models, adopting the approach described above for the national estimates. Here, 5-years of pre-pandemic monthly recorded ASB for the single force were used, drawn from the national open-source data.

## Results

The findings of the ARIMA modelling of police-recorded ASB trends, and of the year-on-year comparison, were presented as Figs. 1 and 2 in the introduction, to set the scene. In the first national lockdown, there was a doubling of recorded ASB which then declined. It remained statistically significantly above expected levels through the first quarter of 2021 but with localised maximums. Across summer 2020, ASB remained around 25 percent above expected levels but rose to 50 percent above during second lockdown, and 75 percent above during third lockdown.

The FOI survey of police recording practices indicated that more than half of forces changed their recording practices to include breaches of COVID regulations as ASB. The format and clarity of responses varied. This excerpt from one force’s response clearly indicates that breaches of COVID regulations were recorded as ASB and that these were also reported as ASB to data.police.uk:

“For COVID breaches, this has been recorded using ASB-Environmental (anti-social behaviour) with ‘free text’ outlining COVID. … [And for reports to data.police.uk] All breaches of COVID reported to the police were recorded as ASB incidents (except for sudden deaths or other incidents that would be a traditional crime)…” (South Wales Police)

Other responses required additional interpretation. This is an excerpt from a different response:

“New opening codes, closure codes and TAGs were used to identify calls relating to COVID-19 breaches…. There were no changes to reporting to www.police.uk.”(Norfolk and Suffolk Constabularies Collaboration)

This response was interpreted as the force guidance not requiring or encouraging breaches in COVID regulations to be recorded as ASB. The reason for this interpretation is that, while new codes and tags were introduced for such breaches, no indication in the response is given that they were recorded as ASB. We note the possibility of interpretation error in relation to some responses and that FOI responses are publicly available for further scrutiny.

**Fig. 3:**
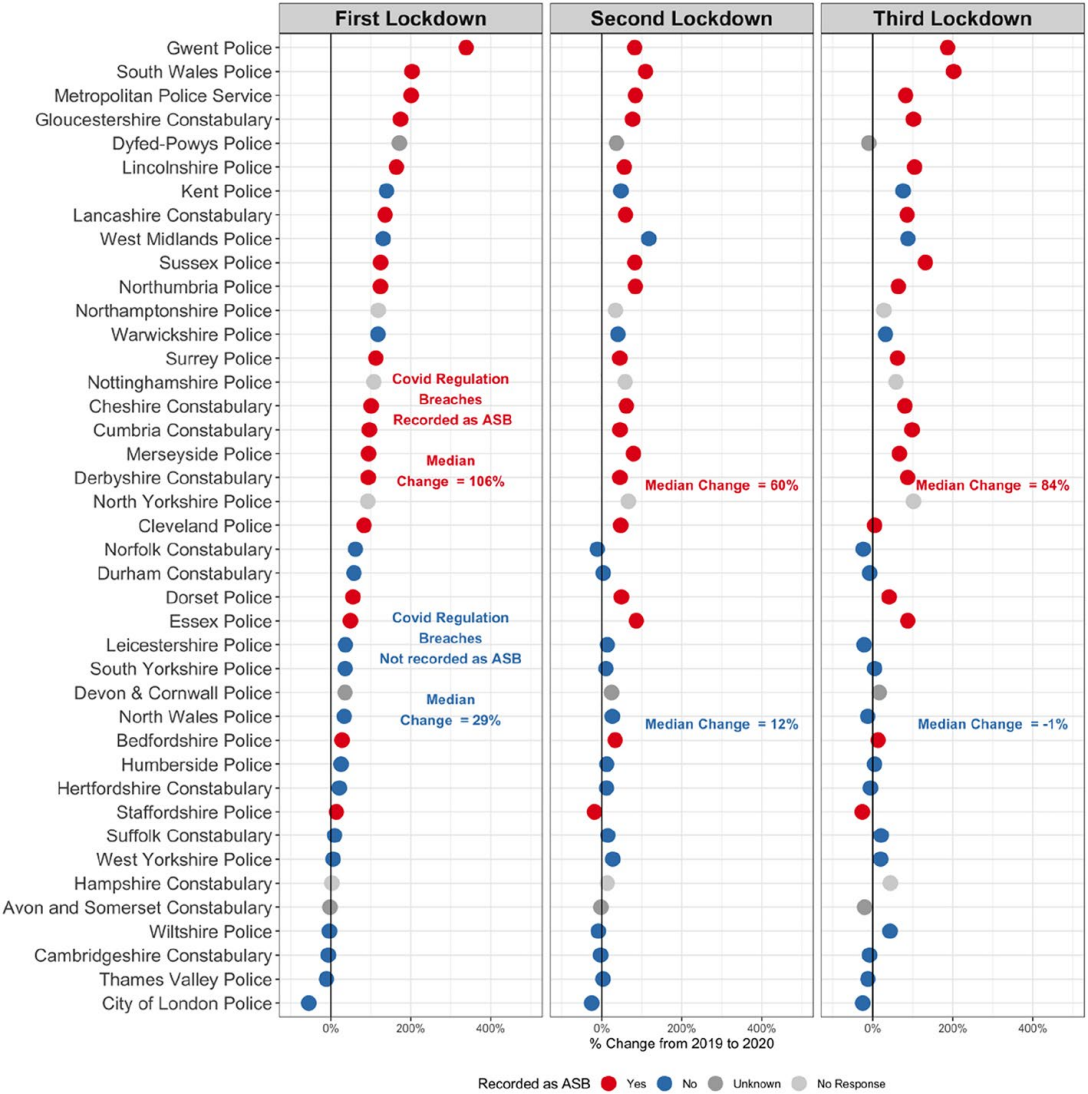
Change in ASB by police force and lockdown

When responses were collated, the survey revealed a strong relationship between force-level ASB increases and the recording of COVID regulation breaches as ASB, as shown in Fig. 3. This is summarised using the respective lockdown medians of 100 percent versus 33 percent, 59 versus 12 percent, and 82 versus 4 percent shown on Fig. 3. There were notable exceptions including Gwent, Kent and West Midlands Police which reported that they did not include breaches of COVID regulations as ASB, but large increases in ASB were recorded nevertheless. When overall ASB rates declined nationally in second lockdown, the force-level change was experienced disproportionately among those forces that included COVID regulation breaches as ASB. By third lockdown, in forces which did not include COVID-regulation breaches as ASB, recorded ASB levels were mostly at levels similar to expected (compared to 2019).

**Fig. 4:**
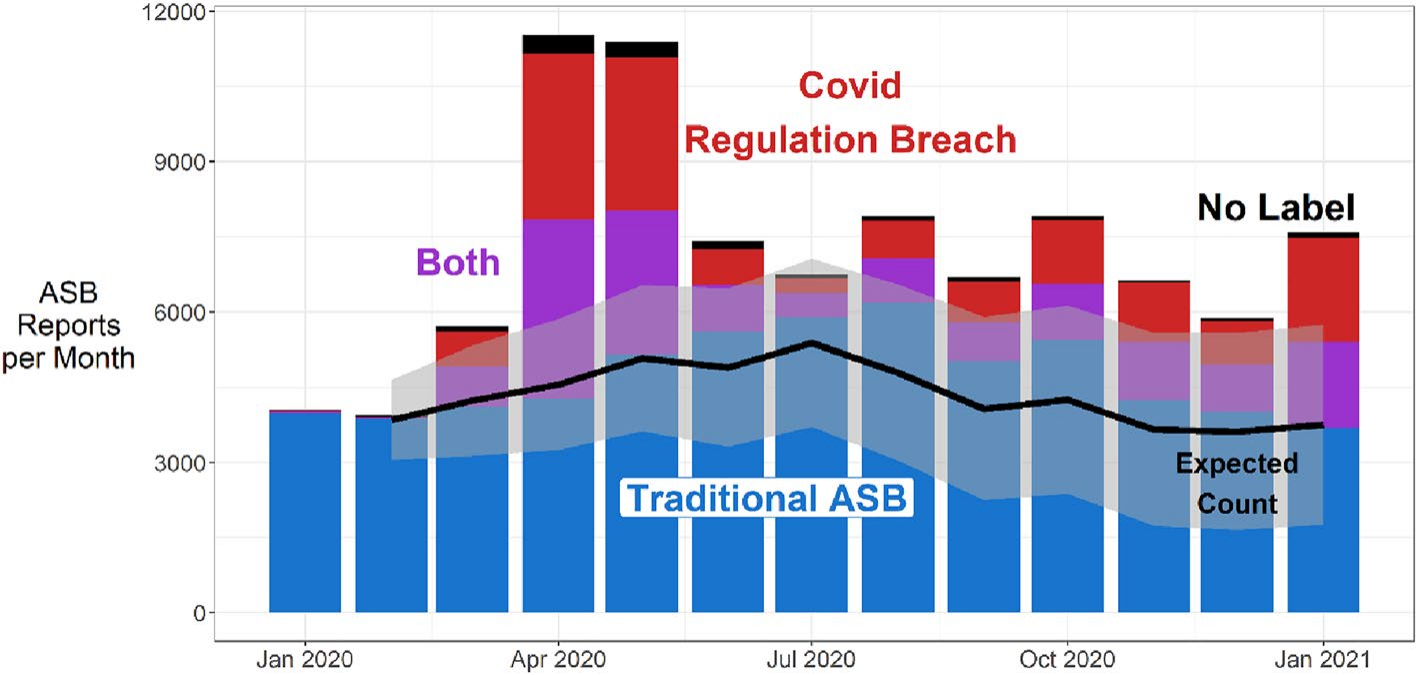
NLP categorisation of ASB

The findings from the NLP of the text contained in ASB incident records in one police force are summarised in Fig. 4. The line of expected ASB was estimated using ARIMA model based on 5-years of pre-pandemic ASB for the single force, and the shaded area around it is the 95 percent confidence intervals. Note that in addition to those incidents categorised as either Traditional ASB or breaches of COVID-regulations recorded as ASB, that there is a third category where both were present. The ASB trend recorded by the force used here for NLP was similar to, but higher than, the national trend. However, this is what would be expected because the national estimates include forces that did not include breaches of COVID regulations as ASB.

The relationship between actual and expected ASB incidents at the force-level is generally similar to that at the national-level (the ratio of actual to expected is higher in the single force, which we explain in the discussion below). Actual ASB recorded was dramatically higher during first lockdown, then smaller but still above expected levels subsequently. Only in July 2020 was the difference between actual and expected not statistically significant (the top of the bar is inside the 95 percent confidence interval).

The NLP categorised all pre-pandemic incidents as traditional ASB. This gives us further confidence in the accuracy of the categorisation. During the pandemic, around half of those incidents not classified as traditional-ASB still contained elements of traditional ASB alongside elements of a breach of COVID regulations.

During first lockdown, traditional-ASB alone almost exceeds the expected upper confidence limit. However, ASB records with some component of breach of COVID-regulations account for all of the increase in ASB incidents during first lockdown. Note that this suggests traditional-ASB did not decline in line with many recorded crime types.

After first lockdown, during the summer of 2020 and particularly in August, traditional-ASB alone exceeded expected levels. From the start of second lockdown in November, traditional-ASB declined to expected levels. The proportion of COVID-related ASB was always much smaller after first lockdown and at its lowest in July–September. There was an increase in ASB relating to breach of COVID regulations in January 2021.

## Discussion

There is consistency in the findings from the FOI survey and the NLP in relation to those aspects where they can be compared. Both suggest that breaches of COVID-related incidents accounted for the bulk of above-expected levels of ASB. Both are consistent with traditional-ASB (excluding breaches) remaining at similar to expected levels across the pandemic.

The NLP component of this study found that effectively all of the recorded ASB increase above expected levels related to breaches of COVID-regulations. However, about half of these were ‘both’ breaches and some form of traditional ASB. The aggregate level of traditional ASB generally stayed at around expected levels. This suggests that it is likely the inclusion of breaches of COVID regulations as a component of police-recorded ASB is largely responsible for the large peak during first lockdown, and for the general increases above expected levels during the first year of the pandemic.

The increase in police-recorded ASB relative to expected levels across the first year of the pandemic, and particularly during first lockdown, was in stark contrast to that of most recorded crime types. The first national lockdown brought the most extensive legal restrictions, resulting in a broader range of breaches relating to movement outside of local areas in particular. Forces with the largest increases in recorded ASB were often those with known beauty spots or coastlines to which many people travelled in breach of regulations. Overall, we suggest that the findings of the study, framed by the changes in COVID regulations, are also thereby consistent with lifestyle theory and the mobility theory of crime in the pandemic.

The FOI survey found that around half of police forces included breaches of COVID-regulations as ASB. A Chief Constable direction, guiding officers to include breaches of COVID regulations as ASB, would be sufficient to change force recording practice. Those police forces which so widened their definition tended to be those reporting larger ASB increases relative to expected levels. The exceptions may warrant further study, and study limitations are discussed further below. Other force-level variation is likely to reflect geographical and other differences such as the prevalence of sought-after beauty spots or coastline to which people travelled during lockdown in breach of regulations, and to reflect how changed lifestyles and movement impact differently by area.

It is clear that police services differed in their ASB recording practices. Perhaps this should not be surprising because the pandemic was a time of significant upheaval and change in policing as in all walks of life. More generally, problems with the quality of police records are a known issue: in 2014 police recorded crime statistics lost their status as national statistics, as defined by the Office of National Statistics, due to concerns over quality (Barrett, 2014; Travis, 2014).

The broad set of behaviours that can potentially be defined as ASB, plus the scope for subjective interpretation in the definition (as discussed earlier), mean that perhaps it should not be surprising if differences of interpretation were compounded in the pandemic. The malleability of ASB’s definition is both a strength and a weakness: it can provide leverage for police and other agencies to deal with unwanted behaviour without resorting to criminal sanctions, but it can lead to ambiguity, disagreement, and on occasion the problemmatic criminalization of otherwise non-criminal behaviour.

The findings of the NLP categorisation of incidents suggests the situation is more nuanced than the simple addition to ASB of COVID regulation breaches. If those incidents recorded as solely breaches of COVID regulations were excluded, there would still have been a dramatic overall increase due to ASB incidents that included an aspect of breach of COVID regulations. Consequently it remains somewhat uncertain whether this reflects an increase in traditional-ASB or a change in reporting behaviour. Consider a person disturbed by a noisy party at night. Pre-pandemic they may have given the revellers some grace, perhaps only calling the police much later in the night if the noise persisted. During the pandemic, a noisy party was also quite likely to be a breach of COVID regulations: this may have increased the likelihood of a call to the police, effectively lowering the threshold (of volume and duration) at which a ‘noisy party’ call is made. Of course, noisy parties are only one type of ASB, and non-local travel during first lockdown was a significant issue. Likewise, the increased opportunity for friction to occur between neighbours, each more likely to be home, may have caused increases in other types of between-neighbour ASB as found in the study of 70 Mexican cities (Hoehn-Velasco et al., 2020).

The study findings allow us to reconcile key differences between police-recorded ASB and that revealed by the telephone CSEW (Office for National Statistics, 2020). The telephone CSEW did not include breaches of COVID-regulations as ASB, though it would include some breaches if there was also traditional ASB. Hence the smaller rise indicated by the telephone CSEW in first lockdown is much more consistent with police-recorded traditional-ASB when COVID-related breaches are excluded. The separate telephone CSEW measures of breaches of COVID-regulations, which were witnessed by around half of respondents, can also be interpreted as consistent with the effect identified in recorded ASB rates.

From March 2020, police were encouraged to enforce COVID regulations using the ‘Four E’s’ approach: (1) *Engage* with people, to ask why they appear to be breaking the rules (2) *Explain* the law, stressing the risks to public health and the NHS, (3) *Encourage* them to change their behaviour and (4) if all else failed, *Enforce* by issuing penalty notices (Brown 2021). Other things equal, the fourth aspect of the Four Es means that fixed penalty notices (FPNs) issued for COVID-related breaches will reflect the more serious breaches of COVID regulations. Evidence published by the National Police Chiefs’ Council (National Police Chief’s Council (NPCC), 2020, 2021) shows that FPN trends in the first year of the pandemic were very similar to those in ASB, with a major peak in first lockdown followed by decline. Given that ASB is likely to have been used as a catch-all category for those breaches of COVID-regulations that did not meet the threshold for issuances of an FPN, the two trends will correlate and the FPN trend serves as indirect verification of our interpretation of the results of the present study.

### Limitations of the study

While the FOI survey achieved an 86 percent response rate, we cannot entirely rule out the possibility of some inaccuracies in responses received. That could occur through misinterpretation of, or misunderstanding, the questions. A series of related questions, formulated by a team including a police practitioner, plus close scrutiny of all responses received, were the primary means of seeking to minimise this possibility. Nevertheless, further research to verify the findings, as well as anomalies (such as police forces with a large increase in recorded ASB but where breaches of COVID-regulations were not included), would be useful.

The NLP analyses drew on ASB incidents from a single police force. That force included COVID-regulation breaches as recorded ASB and experienced similar overall trends to the national level across the pandemic (with the higher rates in the single force also explained). Hence while cautious extrapolation to the national level is reasonable, we recognise that further research is required to address representativeness more unequivocally. Extending the NLP analysis nationally is one possibility that would also allow further testing of the NLP classifications identified here.

Natural Language models can be powerful but rely on the written data that is recorded. When labelling the data, the research assistants were required to make an informed decision, and the training and research protocols sought to minimise discretion (see Appendix 2). The initial recording of incidents by police call handlers was outside the control of the present study however. As discussed in the introduction, ASB is a mixture of behaviours, and the present study was unable to delve more deeply into particular types, so other changes to the composition of ASB were not observed here. Further work on this, and on using NLP to study police-recorded text is likely to be productive.

### Conclusion

The study allows us to answer the key research questions: Why were recorded ASB trends so different from those in most types of recorded crime? Why were police-recorded ASB trends so different to these indicated by the telephone CSEW? The sharp increase in recorded ASB in first lockdown was primarily due to breaches of COVID-regulations that were recorded as ASB. Around half of the increase also involved some form of traditional ASB. Note that this means, even when breaches of COVID regulations are discounted, traditional ASB recorded by police did not decline in a similar fashion to most recorded crime types.

The study findings allow us to reconcile our understanding of the relationship between recorded ASB trends and the distinctly different patterns identified in many types of recorded crime. The recorded crime types that experienced major declines during lockdown had previously been located disproportionately in inner-city urban areas where human presence and interaction decreased dramatically (Halford et al., 2020, Langton et al. 2021a2021a, b). When the inclusion of breaches of COVID regulations are considered, the different changes are, we suggest, all consistent with changes to lifestyles and movement that occurred.

In England and Wales, the first lockdown was the most restrictive: the volume of COVID regulation breaches declined thereafter, reflecting the reduced intensity of subsequent lockdown laws. At the time of finalising this manuscript, there is evidence that ASB levels returned to expected levels during 2022 (see Dixon et al. 2022). A preliminary interpretation is that this is consistent with the present study, reflecting reduced COVID-regulation breaches as the major restrictions on behaviour were removed. Thus the return to expected levels of recorded ASB in 2022 arguably provides indirect support for the present study’s findings.

This study found variation in police recording practices. Around half of forces self-reported including breaches of COVID-regulations as ASB. Those forces tended to also record the largest increases in ASB. If all forces had included such breaches as ASB then it is reasonable to expect the increase in recorded ASB would have been greater. However, if no forces had included breaches of COVID regulations as ASB then the more modest overall increase in ‘traditional ASB’ and ‘both’ breaches and traditional, we postulate, would still have been visible in the national trend.

The present study offered what we believe to be the first application of NLP for the interrogation of the text in police incident log records. Our use of NLP was limited to a particular geographic area and problem, but thereby serves as proof of concept, which we suggest may have the potential for additional and alternative applications in policing globally.

## Data Availability

All data is open source with the exception of that retrieved from Lancashire Constabulary. Restricted data can be made available on request, subject to approval from the originating police service. National ASB data was freely available from data.police.uk. All forces are required to make FOI responses publicly available. The ASB data from the single force was restricted.

## Appendix 1

### Freedom of information survey

#### Full FOI request

‘To whom it may concern,

We are researching the rise in Anti-social behaviour during 2020 and how it is connected with the pandemic in general and lockdowns in particular.

In particular we believe that part of the increase in ASB is down to reports of COVID-19 rule infringements—but as the recording differed across forces then the national figures (from data.police.uk) only tell part of that story.

For our research we are utilising the data.police.uk data and so we would like to know how reporting of lockdown breaches, social distancing infringements and other complaints were recorded by your force and how that will relate to the reporting you do for data.police.uk.

Our understanding is that different forces recorded reports of COVID rule infringements in different manners. Some forces recorded all reports of infringements as Anti-social behaviour and so their ASB figures have risen correspondingly. Whereas other forces recorded infringement reports as a separate category altogether and so that may not have been recorded through data.police.uk whatsoever.

Our questions are therefore:

Generally, during the pandemic how have you recorded within the control room incoming reports relating to COVID-19 breaches?

How did this recording practice manifest, either directly or indirectly, to data.police.uk reporting?

Did your Force record reports of COVID-19 rule infringements as ASB?

Did your Force use an existing category for COVID-19 rule infringement other than ASB?

Did you Force record reports of COVID-19 rule infringements as another category that is reported to data.police.uk?

If you did use an alternate incident class such as ASB, did you apply any ‘tagging’ system to capture the COVID infringements within recorded incidents?

If you did use a tag what tag labels did you use?

Did your Force create a new category for recording reports of COVID-19 infringements?

If so is this new category reportable to data.police.uk?

Thank you for your time.

## Appendix 2

### Natural language processing

The data was the text recorded for each incident closed as ASB. This could vary from semi-structured information, such as that from a log on the online reporting system, to unstructured and unedited text entered as the incident progresses. As part of the research Data Management Plan, data underwent a whitelisting process, to remove personal information, before release. A list of common words, but not names, comprised the whitelist: words on the list were released and others replaced by ‘xxxxx’. So for instance “Bob hit the victim” would become “xxxxx hit the victim” to the research team. While the whitelisting could influence model accuracy, this could not be measured for present purposes because it would require comparison of the pre- and post-whitelist datasets of which only the latter was available for research purposes.

Two student researchers were employed to label data. Before labelling, they were trained on what to look for and how to label. Training was conducted both a former police Detective Chief Inspector (EH) and the researcher undertaking the NLP (AD). The student researchers labelled each item independently of each other. Their interpretations were compared and disagreements adjudicated by the research team.

Four data sets were iteratively constructed. Validation and test data (200 incidents in each) were randomly selected from the complete set of incident records. The validation set was used in the modelling process to inform the adjustment of model parameters and gauge model success as it was trained. The training set was used to quantify the final model after selection. The training data was the third dataset. This was built through active learning (Settles, 2009) whereby the first selection is random, but as the data set grows the data is selected by how unsure the model is about the classifications for that particular incident text. The most difficult texts were labelled to provide the model with hard-to-classify examples. The fourth data set was all data remaining after removal of the other three sets (500 incidents).

The pre-trained language model used the transformer architecture (Vaswani et al., 2017) and BERT, a reputable and popular model (Devlin et al., 2019). The downside of these models here is that they are quadratic in their computation requirements with respect to text length. For that reason, BERT was limited to texts of 512 tokens. Since some of the records had up to 2000 tokens, the Longformer model (Beltagy et al., 2020) was used because its transformer arrangement scales linearly with text length. Even so, the computational requirements were relatively high and the analysis environment was restricted at 32 GB memory. This meant that model parameter selection was partially constrained by memory available. The main constraint was a token limit of 1500, with any excess trimmed from the small number of longer records. The model operated in Python 3.8 using the transformers package on a Pytorch framework. The model was trained 10 times, and validation data results recorded: the model with the best results was used on the test data to quantify performance and on all ASB records to produce the required labels.

The Matthews Correlation Coefficient (MCC) was chosen as the overall measure because the data classes were imbalanced in the dataset (Chicco & Jurman, 2020). An MCC of 1 would be a perfect model with every classification correct, and an MCC of 0 equivalent to random selection. Our breach of COVID regulation model achieved an MCC of 0.74 and the ‘traditional ASB’ model an MCC of 0.68. The models are imperfect but these scores are comparable to those achieved by the original Longformer architecture on benchmark NLP tasks (Beltagy et al., 2020).

This NLP of ASB incident logs had recognized limitations in the data (due to whitelisting) and the model (due to memory constraints). Error analysis could not be conducted because limitations on memory prevented the use of explainable AI techniques such as LIME. Nevertheless, the base model proved relatively robust and produced a trained model with respectable metrics. This indicates that the model performs acceptably as a means of identifying incident types, such that the results are representative of aggregate patterns in the incident data.

See Table

**Table 3:** Model confusion matrices generated from test data with final models

	Labelled as traditional ASB	Not labelled as traditional ASB		Labelled as breach of COVID regulation	Not labelled as breach
Traditional ASB	166	7	Breach of COVID regulation	36	6
Not traditional ASB	7	18	Not breach	12	144

^3^.

## Footnotes

1 https://www.gov.uk/civil-injunctions-criminal-behaviour-orders. Accessed 10 March 2022.

2 ‘High or fairly high level ASB’ is a composite measure derived from responses on seven individual types of ASB as described in chapter 5 of the user guide (ONS 2021a).

3 Table S34 of the ONS supplementary CSEW tables for the year ending March 2020, at https://www.ons.gov.uk/peoplepopulationandcommunity/crimeandjustice/datasets/crimeinenglandandwalesannualsupplementarytables.

4 UK population statistics by country for mid-2020 from the Office for National Statistics at: https://www.ons.gov.uk/peoplepopulationandcommunity/populationandmigration/populationestimates/bulletins/annualmidyearpopulationestimates/latest.

5 A useful summary timeline of lockdowns and measures is available at https://www.instituteforgovernment.org.uk/charts/uk-government-coronavirus-lockdowns. Accessed 11 March 2022.

6 https://www.gov.uk/government/news/rule-of-six-comes-into-effect-to-tackle-coronavirus. Accessed 11 March 2022.

7 https://www.gov.uk/government/publications/covid-19-response-living-with-covid-19. Accessed 11 March 2022.

8 Fixed penalty notices could be issued for breaches of a range of covid regulations: https://publications.parliament.uk/pa/jt5801/jtselect/jtrights/1364/136405.htm. Accessed 11 March 2022.

9 www.data.police.uk.
